# Comparative Analysis of Hot and Cold Brews from Single-Estate Teas (*Camellia sinensis*) Grown across Europe: An Emerging Specialty Product

**DOI:** 10.3390/antiox12061306

**Published:** 2023-06-20

**Authors:** Patricia Carloni, Alfonso Albacete, Purificación A. Martínez-Melgarejo, Federico Girolametti, Cristina Truzzi, Elisabetta Damiani

**Affiliations:** 1 Department of Agricultural, Food and Environmental Sciences-D3A, Università Politecnica delle Marche, Via Brecce Bianche, I-60131 Ancona, Italy; p.carloni@univpm.it; 2 Centro de Edafología y Biología Aplicada del Segura, Agencia Estatal Consejo Superior de Investigaciones Científicas (CEBAS-CSIC), Department of Plant Nutrition, Campus Universitario de Espinardo, E-30100 Murcia, Spain; alfonsoa.albacete@carm.es (A.A.); pmelgarejo@cebas.csic.es (P.A.M.-M.); 3 Department of Life and Environmental Sciences, Università Politecnica delle Marche, Via Brecce Bianche, I-60131 Ancona, Italy; f.girolametti@univpm.it (F.G.); c.truzzi@univpm.it (C.T.)

**Keywords:** *Camellia sinensis*, single-estate European teas, tea varieties, hot and cold brews, total polyphenol content, antioxidant profile, UV-Vis spectroscopy, metabolomic profiling

## Abstract

Tea is grown around the world under extremely diverse geographic and climatic conditions, namely, in China, India, the Far East and Africa. However, recently, growing tea also appears to be feasible in many regions of Europe, from where high-quality, chemical-free, organic, single-estate teas have been obtained. Hence, the aim of this study was to characterize the health-promoting properties in terms of the antioxidant capacity of traditional hot brews as well as cold brews of black, green and white teas produced across the European territory using a panel of antioxidant assays. Total polyphenol/flavonoid contents and metal chelating activity were also determined. For differentiating the characteristics of the different tea brews, ultraviolet-visible (UV-Vis) spectroscopy and ultra-high performance liquid chromatography coupled with high-resolution mass spectrometry were employed. Overall, our findings demonstrate for the first time that teas grown in Europe are good quality teas that are endowed with levels of health-promoting polyphenols and flavonoids and that have an antioxidant capacity similar to those grown in other parts of the world. This research is a vital contribution to the characterization of European teas, providing essential and important information for both European tea growers and consumers, and could be of guidance and support for the selection of teas grown in the old continent, along with having the best brewing conditions for maximizing the health benefits of tea.

## 1. Introduction

Tea, one of the most important cash crops in the world, is second only to water as the most popular beverage worldwide and is produced from the combination of cured leaves and buds of the *Camellia sinensis* (tea) plant with hot and, more recently, with cold water. It is generally perceived as a healthier drink than carbonated ones and coffee, and the wide spectrum of health benefits associated with tea consumption such as weight loss, cardiovascular health, neuroprotection, antiaging, anti-inflammatory, antioxidant, antimicrobial and antidiabetic activities are reviewed in [1]. These health-promoting properties have led to a rise in the demand for green and black teas in the European tea markets, which is due also in part to assertive marketing and campaigns showcasing tea’s health benefits. Indeed, the European tea market is currently witnessing a premiumization trend with the market expected to reach a compound annual growth rate (CAGR) of 4.9% between 2023 and 2028 [2]. This is also driven by the rise in popularity across Europe of individual, gourmet, high-quality specialty teas with unique flavors. Although the market for these specialty teas in Europe is still relatively small (experts estimate it to be 5%) compared to commodity teas, it is nonetheless a rapidly growing market. In the specialty tea segment, as dedicated consumers become more interested and more knowledgeable about high-quality tea, more attention is being given to the different tea varieties, their origin and their proper brewing techniques, especially amongst Millennial and Baby Boomer generations [3]. For example, staunch consumers monitor brewing time and have learned that for some teas water temperature and water quality influence the in-cup tea experience. As a result, trendy teapots and kettles designed specifically for brewing leaf tea and controlling water temperature are finding their way into the market. This, thus, opens up greater chances for suppliers of specialty teas. 

Tea is grown around the world under extremely diverse geographic and climatic conditions, namely, in China, India, the Far East and Africa. China and India are in the lead for green and black tea, respectively, with China accounting for 46.6% of world tea production, with an output of 2.93 million tons in 2020, and India produced 1.26 million tons [3] in the same year. However, growing tea also appears to be feasible in many regions of Europe, although tea cultivation is not typical of Europe. Over the last 150 years, there have been a number of experiments in Europe for growing the *Camelia sinensis* plant and making tea from it, with the earliest experiments being found in Portugal (the Azores islands) and France [4]. Around 15 years ago, several individual initiatives were launched in various European countries, namely, France, Germany, Italy, Holland, Portugal, Spain, Switzerland and the United Kingdom, and in some cases, the experimental stage has been passed successfully, and the “pilot production” is ongoing. This led to the creation of the Association “Tea Grown in Europe” (EuT) in 2016, whose objective is to promote, in Europe, an economic activity domain based on the cultivation of tea plants on micro-plantations of the European territory [5]. This domain encompasses the whole value chain, from cultivation to transformation in marketable products; in this case, in high-quality, chemical-free, organic, single-estate teas from Europe that focus on sustainable practices. To date, however, no studies have been undertaken to characterize the antioxidant profile of these novel European specialty teas that have been grown under diverse pedoclimatic (soil type, sun exposure, rainfall) conditions and from different cultivars. These factors are known to influence the contents of catechins, the main tea polyphenols that are correlated with antioxidant capacity [6]. The degree of oxidation of the tea leaves during processing provides different tea categories and also influences the antioxidant capacity, with the unoxidized (non-fermented) green and white teas usually displaying a higher antioxidant profile than the semi-oxidized (oolong) and oxidized (fermented) black teas [7].

**Figure 1:**
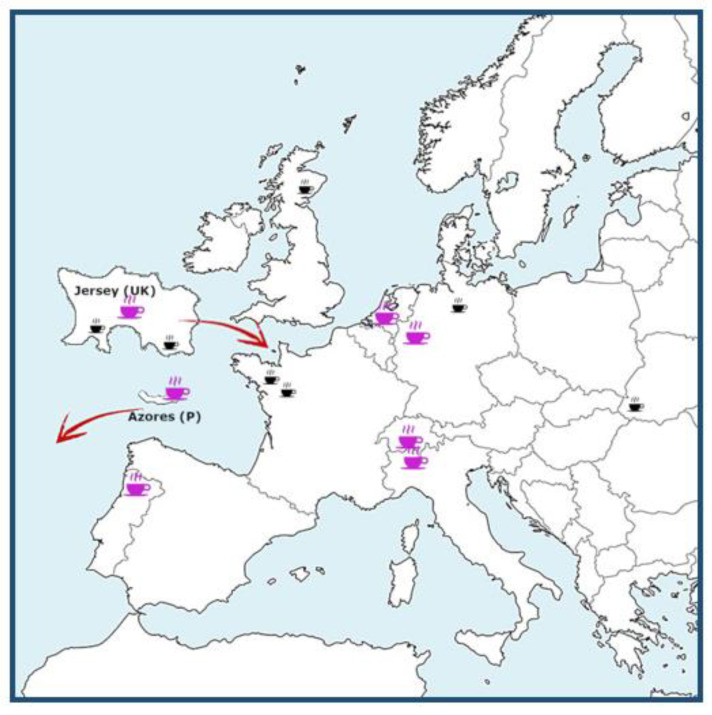
Graphical distribution of tea micro-plantations across Europe (teacups). The fuchsia teacups indicate the origin of the teas that were studied in this work.

With this background, the aim of the present study was to characterize the health-promoting properties in terms of the antioxidant capacity of black, green and also white teas produced across the European territory from members of EuT (Figure 1) using a panel of antioxidant assays, as well as determining their total polyphenol and flavonoid content and metal chelating activity. 

In this study, besides the traditional hot brew, a cold brew was also employed since cold tea is a popular thirst-quenching beverage in the summer months. For differentiating the characteristics of the different tea brews, ultraviolet-visible (UV-Vis) spectroscopy was employed, whereas metabolomic profiling of bioactive compounds was carried out using high-resolution mass spectrometry on the hot, green tea infusions only. The overall information obtained is essential to pave the way for European teas in the global tea market, and it is of importance for both growers and consumers to know what a cup of European tea has to offer.

## 2. Materials and Methods

### 2.1. Chemicals and Equipment

The chemicals that were used were all purchased from Merck KGaA (Darmstadt, Germany): [2,2′-azinobis-(3-ethylbenzothiazoline-6-sulfonic acid) diammonium salt] (ABTS), 6-hydroxy-2,5,7,8-tetramethylchroman-2-carboxylic acid (TX), gallic acid (GA), Folin-Ciocalteu reagent (2N solution), potassium persulfate (K_2_S_2_O_8_), sodium carbonate (Na_2_CO_3_), 3′,6′-dihydrosyspiro[isobenzofuran-1[3H],9′[9H]-xanthen]-3-one (fluorescein), [2,2′-azobis(2-methylpropionamidine) dihydrochloride] (AAPH), 3-(2-pyridyl)-5,6-diphenyl-1,2,4-triazine-4′,4′′-disulfonic acid sodium salt (Ferrozine), iron(II) sulfate heptahydrate, (+)-catechin hydrate, sodium nitrite (NaNO_2_), aluminum chloride hexahydrate (AlCl_3_ • 6H_2_O), sodium hydroxide (NaOH), 2,4,6-tripyridyl s-triazine (TPTZ), iron(III) chloride (FeCl_3_), ascorbic acid (AA), sodium acetate (CH_3_COONa), acetic acid (CH_3_COOH), hydrochloric acid (HCl), potassium dihydrogen phosphate (KH_2_PO_4_), dipotassium hydrogen phosphate (K_2_HPO_4_) and ethanol absolute RPE grade. Ultrapure water was generated from a Milli-Q system by Merck Millipore (Merck KGaA, Darmstadt, Germany) and was used for all the experiments. Mineral water ACQUA SANT’ANNA S.p.A. (Vinadio, CN, Italy) with a fixed residue at 180 °C of 22 mg/L and total hardness of 0.98 °f used for tea infusions, was purchased from the local supermarkets. Spectrophotometric Vis measurements were recorded in triplicate on a microplate reader (Synergy HT, Biotek, Winooski, VT, USA).

### 2.2. Tea Samples

Thirteen tea samples coming from seven gardens located across Europe (Figure 1), whose leaves had been harvested in the 2021 season, were studied. The tea growers and members of EuT who decided to participate in this study are the following: Compagnia del Lago Maggiore (Italy), Het Zuyderblad (The Netherlands), Casa del Tè Monte Verità (Switzerland), Tschanara Teagarden (Germany), Jersey Fine Tea (UK), Chà Camèlia (Portugal) and Agrarian Devt. Services Sao Miguel, Azores (Portugal). Details regarding the temperature range of these gardens, location, growing season, altitude, humidity and average rainfall can be found in the EuT association leaflet [5]. The tea samples are described in Table 1, where they are identified with an acronym of two letters indicating the country of origin, Italy = I, The Netherlands = N, Switzerland = S, Germany = G, Jersey (UK) = J, Portugal = P and Azores (P) = A, followed by the type of tea: green = G, black = B and white = W. Furthermore, for the infusions, a letter indicating the type (C= old, H = hot) was added at the end as reported in the tables and figures in the Results and Discussion sections.

Six tea samples were green (G), five were black (B) and two were white (W); they had been processed in their respective gardens following acceptable guidelines of the tea practice industry, starting from hand-plucked, fresh tea shoots (usually one or two leaves and a bud) [8]. The general processing steps for the above teas are outlined as follows: the black tea was produced through the withering of the tea leaves followed by hand-/electric-rolling, fermentation/aeration and drying; the green tea was produced through heat enzyme inactivation of the leaves (steaming or wok-/pan-firing) followed by hand-/electric-rolling and drying; white tea was simply produced through withering and drying.

### 2.3. Preparation of Tea Brews

Two different brews were prepared from each sample using, in each case, 1.0 g of tea leaves and 50 mL of mineral water, representing the average weight of plant material contained in a tea bag or in a heaped teaspoon and the average volume of a cup of tea [9]. Mineral water rather than tap water was used since its composition is stable and known. Prior to the preparation of infusions, the tea leaves were milled using a hand-mill to obtain a homogeneous fine powder for each kind of tea in order to reduce the variability in extraction efficiency arising from leaf size. The cold brews (C) were prepared by pouring mineral water at room temperature (20–25 °C) over the tea powder in a glass jar and gently agitating for 5 s. The jar was then closed, and the infusion was left to stand in the refrigerator (4–6 °C) for 16 h. For hot (H) tea infusions, mineral boiling water (95–100 °C) was poured over the tea powder and the infusion was brewed for 5 min. Both hot and cold brews were then filtered through Whatman No. 4 filter paper, aliquoted and stored at −20 °C until analyzed. Each brewing method was performed in triplicate for each sample.

### 2.4. Determination of Total Phenolic Content (TPC) 

The total phenolic content in the tea infusions was determined using the Folin-Ciocalteu reagent [10,11]. Briefly, 50 μL of each tea sample diluted 30 times were transferred into wells of a transparent 96-well microplate to which 150 μL of a 10-fold diluted aqueous solution of Folin-Ciocalteu reagent was added followed by the addition, after 10 min incubation in the dark, of 100 μL 10% *w*/*w* Na_2_CO_3_ aqueous solution. Samples were then incubated at room temperature in the dark for 120 min before measurement at 760 nm against water as blank. The results are expressed as mM Gallic acid equivalents (mM GAEq), using the linear regression value calculated from a gallic acid calibration curve (final concentrations in water: 0.05–0.60 mM) that was run during the assay at the same time as the tea samples. 

### 2.5. Total Flavonoid Content (TFC)

The total flavonoid content in the tea infusions was measured using a colorimetric assay according to the method of Kim et al. with some modifications [12]. Briefly, 50 μL of each tea infusion diluted 10 times was added to each well of a transparent 96-well microplate containing 150 μL of water. The following solutions were then added in the order reported and at the defined times: 12 μL of 5% *w*/*w* NaNO_2_ solution; after 5 min, 12 μL of 10% *w*/*v* AlCl_3_ aqueous solution; after 1 min, 80 μL of 1 M NaOH aqueous solution. After 15 min incubation at room temperature in the dark, the absorbance was read at 510 nm against water as blank. The results are expressed as mM catechin equivalents (mM CEq), using the linear regression value calculated from a catechin calibration curve (final concentrations in water: 0.02–0.35 mM) that was run during the assay at the same time as the tea samples. 

### 2.6. Determination of In Vitro Antioxidant Capacity (ABTS, FRAP, ORAC)

The in vitro antioxidant capacity was evaluated by means of three different methods, namely, Oxygen Radical Absorbance Capacity (ORAC), ABTS and Ferric Reducing Antioxidant Power (FRAP) assays [13,14,15]. 

#### 2.6.1. ORAC Assay

For the ORAC assay, in each well of a solid black 96-well microplate, 50 μL of each tea infusion diluted 800 times with PBS (phosphate buffer saline 75 mM, pH 7.4) was mixed with 160 μL of 0.010 μM solution of fluorescein in PBS. After 10 min incubation in the dark at 37 °C, 90 μL of 25 mM AAPH solution in PBS was rapidly added to each well, and fluorescence was recorded from the top every 120 s for 3 h using an excitation wavelength of 485/20 nm, emission filter of 528/20 nm and constant temperature of 37 °C. The reaction kinetics was typical of classic fluorescence decay due to bleaching of fluorescein that was delayed in the presence of tea samples or of Trolox used as standard. The AUC (area under the fluorescence decay curve) was automatically calculated by the analytical software Gen5 2.00.18 (Biotek, Winooski, VT, USA) integrated into the Synergy HT microplate reader. The net AUC for each standard/compound was obtained by subtracting the area of the control sample that lacked antioxidants. The antioxidant capacity is expressed as mM Trolox equivalents (mM TXEq), using the linear regression value calculated from the Trolox calibration curve (final concentrations in PBS: 0.01–0.08 mM) that was run during the assay at the same time as the tea samples.

#### 2.6.2. ABTS Assay

A stock solution of the colored radical cation (ABTS•^+^) was prepared by mixing a 7.0 mM aqueous ABTS solution with a 24.5 mM aqueous solution of potassium persulfate as the oxidizing agent in a 9:1 ratio, respectively, and allowing the mixture to stand at room temperature in the dark for 12–16 h before use. This stock solution was then diluted ≃50-fold with water to reach an absorbance of 0.9 ± 0.1 at 734 nm. For this assay, 30 μL of each brew previously diluted 120 times with water was added in each well of a transparent 96-well microplate, followed by 270 μL of the diluted ABTS•^+^ solution. The microplate was shaken and left to stand for 120 min at room temperature in the dark before measuring the absorbance at 734 nm against water as blank. The antioxidant capacity was determined as inhibition percentage and is expressed as mM Trolox equivalents (mM TXEq), using the linear regression value calculated from the Trolox calibration curve (final concentrations in water: 0.005–0.250 mM) that was run during the assay at the same time as the tea samples.

#### 2.6.3. FRAP Assay

For the FRAP assay, the working reagent was prepared by mixing immediately before using the following reagents in a 5:5:50 ratio, respectively: 10 mM TPTZ solution in 40 mM HCl, 20 mM FeCl_3_ in water and 300 mM acetate buffer, pH 3.6. This reagent (250 μL) was then added to 50 μL of each brew previously diluted 40 times with water that was present in each well of a transparent 96-well microplate. The microplate was shaken and incubated for 30 min at room temperature in the dark before absorbance measurement at 600 nm. The results are expressed as mM ascorbic acid equivalents (mM AAEq), using the linear regression value calculated from the ascorbic acid calibration curve (final concentrations in water: 0.01–0.60 mM) that was run during the assay at the same time as the tea samples.

### 2.7. Metal Chelating Assay 

The metal chelating activity of the different infusions was determined on ferrous ions according to [16]. Briefly, 50 μL of each brew (undiluted), or of a 1.2 mM EDTA standard aqueous solution appropriately diluted or water as control and blank, was added in each well of a transparent 96-well microplate, followed by 50 μL of a 0.5 mM ferrous sulfate solution (or water for blank) and 200 μL of a 0.25 mM ferrozine aqueous solution (or water for tea blanks). The microplate was shaken and left to stand for 10 min in the dark at room temperature. The absorbance was then read at 562 nm against the corresponding blank (containing water instead of ferrozine for each tea sample and water instead of Fe(II) for EDTA and the control). The chelating activity was determined as an inhibition percentage and is expressed as mM EDTA equivalents (mM EDEq), using the linear regression obtained from the EDTA calibration curve (final concentrations in water: 0.20–0.80 mM) that was run during the assay at the same time as the tea samples.

### 2.8. UV-Vis Spectrophotometric Measurements 

To obtain further information that could differentiate the different tea brews based on their color, spectrophotometric measurements in the UV-visible range were taken [17]. Briefly, 100 μL of each tea infusion were added in each well of a transparent 96-well microplate and the absorbance spectrum (300–700 nm) was recorded at constant intervals (Δλ = 5 nm) against water as blank. The results are expressed as AU (arbitrary units). The obtained data were statistically elaborated setting wavelengths (nm) in the range of 320–500 as X variables, and the corresponding absorbances values as Y variables. Principal component analyses (PCA) were conducted to identify the set of ‘best discriminating’ k variables between groups of tea samples (different types and different infusions) responsible for distinguishing the color differences of the infusions.

### 2.9. Metabolomic Profiling

Extracts from green tea hot brews were filtered through 13 mm diameter Millex filters with a 0.22 µm pore size nylon membrane (Millipore, Bedford, MA, USA). Ten microliters of filtered extract were injected using ultra-high performance liquid chromatography (UHPLC, Accela Series, ThermoFisher Scientific, Waltham, MA, USA) coupled to a high-resolution mass spectrometry (Exactive, ThermoFisher Scientific, Waltham, MA, USA) consisting of an Orbitrap detector and using a heated electrospray ionization (HESI) interface. Data processing was carried out through the Xcalibur software (version 4.3, ThermoFisher Scientific, Waltham, MA, USA), the XCMS metabolomics platform (Scripps Center for Metabolomics and Mass Spectrometry, La Jolla, CA, USA) and the KEGG, PubChem and PHENOL-EXPLORER chemical databases, amongst others. For the fine-tuning analysis method, the molecular formulas of the compounds were searched in the PubChem platform and entered into the Qual Browser package of the Xcalibur software, where mass/charge (*m/z*) ratios of each metabolite were identified in the negative mode, adjusting a mass tolerance of ≤2 ppm in the Processing Setup Package. Additionally, correlations between compounds of the same metabolic pathway and the LogP coefficient were used to accurately identify these metabolites.

### 2.10. Data Analysis

The results of the TPC, TFC, ORAC, ABTS and FRAP tests and metal chelating activity are expressed as mean values with standard deviation (SD) from at least three independent experiments each performed on each of the three prepared infusions (n = 9). 

Statistical differences were obtained through an analysis of variance (ANOVA), followed by a Tukey’s multiple comparison test at a 95% confidence level (*p* ≤ 0.05). The results of the spectrophotometric measurement for color analysis were also processed using multivariate chemometric techniques involving principal component analysis (PCA). These statistical treatments were performed using XLSTAT software (Addinsoft SARL, Paris, France). 

For metabolomic analysis, PCA and partial least squares-discriminant analysis (PLS-DA) were performed using SPSS for Windows (Version 25.0, SPSS Inc., Chicago, IL, USA). The Varimax rotation method was used for the loadings-PCA while score-PCA was graphically plotted as a Bi-Plot score.

## 3. Results 

### 3.1. Total Polyphenol and Flavonoid Contents in Brews from Tea Grown in Europe 

**Figure 2:**
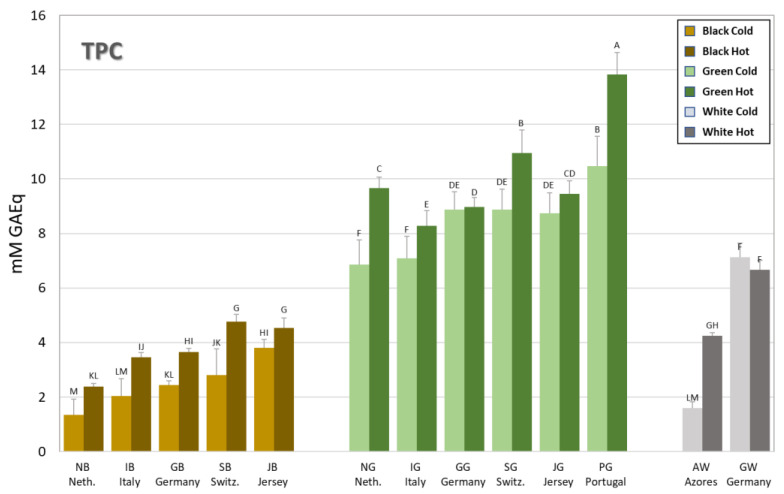
Total polyphenol content (TPC) of the tea brews measured using Folin-Ciocalteu’s reagent. Bars are colored according to the type of tea (brown = black tea; green = green tea; grey = white tea) and the type of infusion (light shade = cold brew; dark shade = hot brew). The letters above the bars indicate the homogeneous sub-classes resulting from Tukey’s post hoc multiple comparison tests (*p* < 0.05). Neth. = The Netherlands; Switz. = Switzerland.

The total content of polyphenols (TPC) in hot (dark color) and cold (light color) brews of teas from European gardens is reported in Figure 2 and Table 2. The results demonstrate that green teas have between 2- and 3-fold higher TPC compared to their black counterparts from each garden. Concerning the type of brew, in most cases, brewing tea in hot water led to a higher and mostly significant TPC than brewing in cold water, regardless of the type of tea. The tea that had the highest significant TPC was the green one from Portugal brewed in hot water (PGH) with a TPC value of 13.8 mM GAEq; all the other green teas scored similar values in the range of 7–11 mM GAEq with no big differences between cold and hot brews but always significantly higher than the corresponding black ones. The black teas had TPC contents in the range of 1.3–4.8 mM GAEq with slight variations amongst the countries and the type of infusion. Greater differences can however be noted amongst the two white teas examined, with the white tea from the German garden displaying TPC values roughly twice (GWC = 7.1, GWH = 6.7 mM GAEq) as high as those obtained from the Azores (AWC = 1.6; AWH = 4.2 mM GAEq). The white tea from the Azores was not significantly different in terms of TPC from the black teas with lower content (NB, IB, GB) when cold brewed and the black teas with higher content in the hot infusion (GB, SB, JB), whereas the one from Germany was not significantly different to the green tea from Italy (IGC) and the Netherlands (NGC) in the cold infusion.

Since the main class of polyphenols present in *Camellia sinensis* is flavonoids, the total flavonoid content (TFC) was also measured in the same cold and hot brews of European teas (Table 2). As would be expected, the trend in the results obtained reflect those of the TPC reported in Figure 2, with a significant correlation obtained using Pearson’s correlation coefficient (Table 3).

### 3.2. Antioxidant Capacity of Brews from Tea Grown in Europe

**Figure 3:**
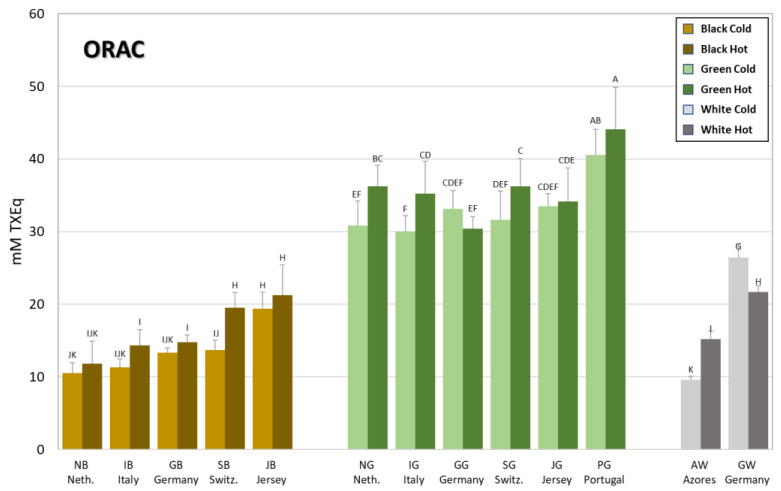
Antioxidant activity of the tea brews measured with the ORAC assay. Bars are colored according to the type of tea (brown = black tea; green = green tea; grey = white tea) and the type of brew (light shade = cold brew; dark shade = hot brew). The letters above the bars indicate the homogeneous sub-classes resulting from Tukey’s post hoc multiple comparison tests (*p* < 0.05). Neth. = The Netherlands; Switz. = Switzerland.

The antioxidant capacity of the hot and cold brews from teas grown in Europe was evaluated using three popular, independent, spectrophotometric assays, ORAC, FRAP and ABTS, in order to generate a more complete antioxidant profile of the different teas that better reflect their potential protective health effects [18,19]. Figure 3 shows the results obtained using the ORAC assay, whereas the results obtained using the other two assays (FRAP and ABTS) are reported in Table 2. From Figure 3 it is clear that green teas display, on average, twice as high antioxidant capacities (30.0–44.1 mM TXEq) than black teas (10.5–21.2 mM TXEq), as would be expected in accordance with their significantly higher (*p* < 0.05) TPC (Table 2). The only outlier is the green tea from Portugal (PG) endowed with the significantly highest antioxidant capacity (> 40 mM TXEq). Concerning the two white teas, the German one (GW) showed a significantly higher antioxidant capacity compared to the white tea from the Azores (AW), reflecting the significant difference (*p* < 0.05) in their respective TPC. In the majority of cases, no statistical differences were observed between the two different brewing methods. However, where statistical differences were observed, it was always the hot brew that displayed a higher antioxidant capacity than the cold one. The only exception was observed for the German white (GW) tea, where this trend was reversed. 

Although the ORAC assay is classified as a direct competition method based on a hydrogen atom transfer (HAT) mechanism, the antioxidant profile trend obtained using this method was similar to those obtained using the other two assays, ABTS and FRAP, which are considered indirect methods based on different mechanisms (HAT and single-electron transfer (SET) for the first one and SET for the latter) [19]. Indeed, the results reported in Table 2 follow a very similar pattern both with regards to the type of tea, green vs. black, and with regards to the brewing method, hot vs. cold, with only a few exceptions. For example, using the FRAP assay, two more teas, namely, the green teas from Jersey (UK), JG, and Germany, GG, resulted in having a statistically significant higher antioxidant capacity when brewed using cold water (JGC = 16.2 and GGC = 17.5 mM AAEq) instead of hot (JGH = 14.2 and GGC = 15.2 mM AAEq). 

This trend in antioxidant profile was confirmed upon examination of the correlations amongst the results obtained from the different assays, including the TPC and TFC ones, obtained using Pearson’s correlation coefficient, as summarized in Table 3. The total polyphenol and flavonoid contents correlate with the antioxidant capacity obtained using the three different assays, with correlation coefficients exceeding 0.96 in all cases, and they were highly significant (<0.0001). This is in accordance with numerous studies demonstrating the close relationship between polyphenols and antioxidant capacity, i.e., polyphenolic compounds, in particular the class of flavonoids, are the main contributors in inhibiting oxidative processes; this is reviewed in [20]. Furthermore, a high correlation exists between the three different assays despite the different chemical principles on which they are based. This indicates that all three assays are equally valid for determining the antioxidant capacity of tea infusions.

### 3.3. Metal Chelating Activity of Brews from Tea Grown in Europe

**Figure 4:**
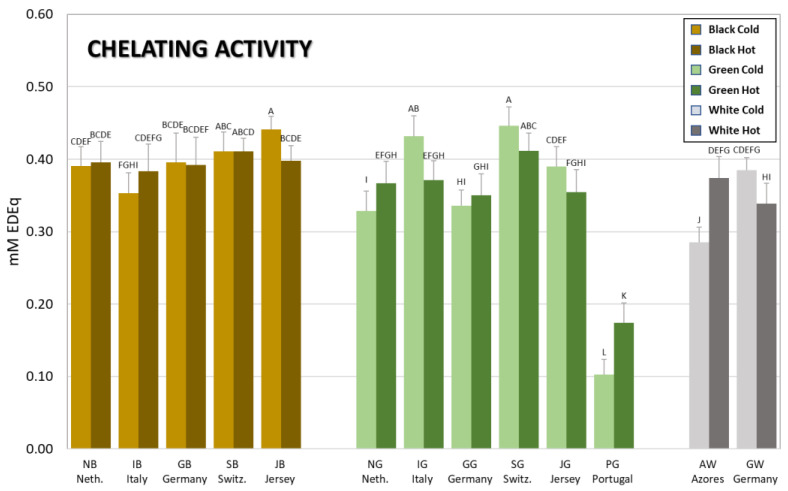
The metal chelating activity of the tea brews measured with the ferrozine assay. Bars are colored according to the type of tea (brown = black tea; green = green tea; grey = white tea) and the type of brew (light shade = cold brew; dark shade = hot brew). The. letters above the bars indicate the homogeneous sub-classes resulting from Tukey’s post hoc multiple comparison tests (*p* < 0.05). Neth. = The Netherlands; Switz. = Switzerland.

The antioxidant capacity of tea brews also depends in part on the propensity of these polyphenol-rich brews to chelate metal ions, which are known triggers of free radical reactions [21,22]. This chelating ability is measured in a competition reaction, whereby polyphenol compounds compete with the compound ferrozine, thus inhibiting the colored complex formed between ferrozine and ferrous ions. Figure 4 shows that overall, unlike the data obtained with the previous assays, no remarkable differences can be observed between the different teas and brewing conditions, which is also indicated by the absence of statistical significance amongst the means of the data obtained (Table 2). The trend, however, shows that black teas have a higher chelating activity than the unfermented green and white teas which are more similar. The only outstanding exception is the data obtained from the green tea from Portugal (PG), which showed a statistically significant, very low, chelating power (PGC = 0.10, PGH = 0.17 mM EDEq) compared to the means of both green (0.34), black (0.40) and white (0.35) teas, regardless of the brewing condition. 

### 3.4. UV-Visible Spectral Characteristics of Brews from Tea Grown in Europe

**Figure 5:**
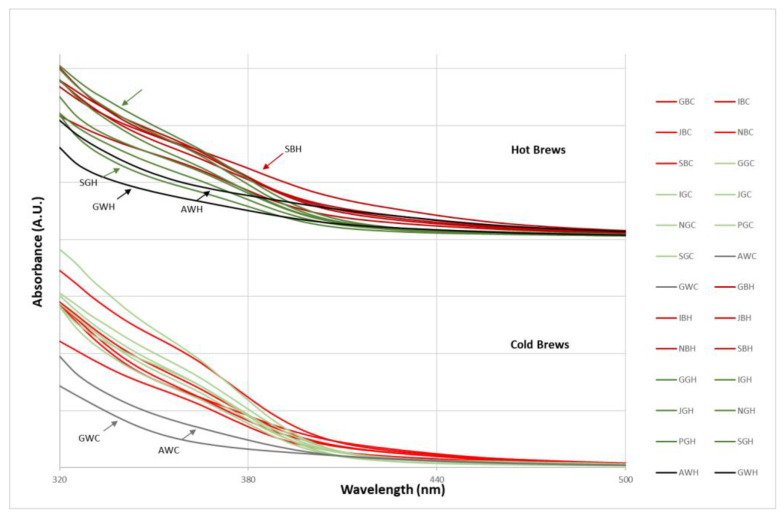
Averaged UV-Vis spectra (320–500 nm, Δλ = 5 nm) of the various cold and hot tea brews. Spectra are colored according to the type of tea (red = black tea; green = green tea; grey = white tea) and the type of infusion (light shade = cold brew; dark shade = hot brew).

The UV-Vis spectra of the various tea brews are shown in Figure 5. No apparent absorption peak could be observed at 300–700 nm due to the complex chemical composition of tea brews, and no absorption above 500 nm was noted. However, amongst all the infusions, white teas had the lowest absorption values in the range of 320–380 nm, with GW differentiating for the lowest absorptions both in cold and hot infusions. Amongst the hot infusions, SB shows the highest absorption in the range of 360–460 nm, while the highest absorption in the range of 320–360 nm is shown by the PG infusion. SG shows, instead, the lowest values amongst the green tea hot infusions, and, generally, all green teas show lower absorptions in the range of 380–440 nm (green lines, Figure 5). This trend can also be observed in the cold infusions. 

**Figure 6:**
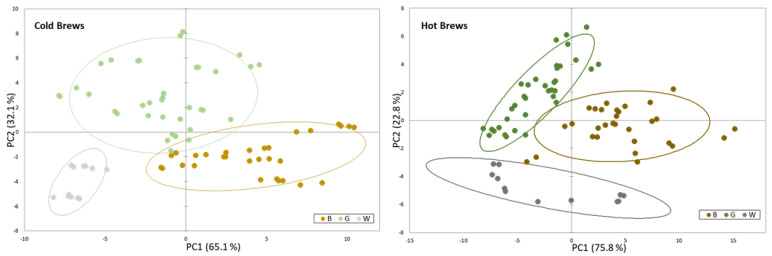
Score plots for the first two components resulting from the principal component analysis performed using the UV-Vis data (320–500 nm, Δλ = 5 nm) of the cold and hot brews. Dots are colored according to the type of tea (brown = black tea; green = green tea; grey = white tea) and the type of infusion (light shade = cold brew; dark shade = hot brew).

To find wavelengths that best discriminate between groups of tea infusions, the UV-visible absorption spectra from 320 to 500 nm of the tea infusions were statistically elaborated. The obtained data were processed using principal component analysis (PCA) as the multivariate data method and with the Pearson correlation coefficient as the index of similarity between variables. The PCA model using the data set of hot and cold infusions treated separately and the different wavelengths (nm) used as variables led in both cases to two significant principal components (PC), with an eigenvalue > 1 that explained a variability greater than 97% of the total system. As shown in Figure 6, the score plots for the first two components clearly discriminated both the hot and the cold infusions, separately elaborated, into three groups containing the infusions of the different types of tea. 

Supplementary Table S1a and b show the eigenvalues, the variance explained and the contribution of the variables (%) for the first two principal components extracted. The first factor PC1 includes wavelengths in the range of 375–500 nm for the cold and 355–500 nm for the hot infusions, while in PC2, the contribution of the wavelengths below 375 nm for cold and 355 nm for hot infusions becomes important.

### 3.5. Metabolomics Analysis of Brews from Tea Grown in Europe

The phenolic composition of the green tea hot brews was evaluated using a high-resolution untargeted UHPLC-MS approach. Only green tea was selected for metabolomic analysis since amongst these teas more significant differences were observed, especially with regards to the Portuguese one, which stood out in terms of TPC, TFC and antioxidant and metal chelating activities. Therefore, it was of interest to examine the differences in the metabolomic profile of these teas. Following normalization and correction of the metabolomics data, 57 compounds shared by all tea origins were putatively annotated, including 25 flavonoids, 21 phenolic acids, and 4 stilbenes (Table S2—Supplementary Material). Some of the major and other minor polyphenols of tea, including (+)-catechin, (-)-epicatechin, (-)-epicatechin 3-O-gallate, theaflavin and gallic acid were also putatively annotated [23]. The samples were found to also be rich in other phenolic acids, such as punicalin, syringic acid, vanillic acid, caffeic acid, ferulic acid, p-coumaric acid, rosmarinic acid and sinapic acid, and anthocyanins such as cyanidin, delphinidin, pelargonidin, peonidin, phloretin and/or their glycosides. 

In addition, considering that the main differences were represented in the metabolomic dataset, a supervised PCA multivariate statistical approach was used to identify the contribution of each (poly)-phenolic compound for discrimination purposes, according to the different gardens where the tea samples were harvested. The PCA analysis performed on the green tea samples prepared in hot water permits the extraction of four significant principal components (PC) with an eigenvalue > 1 that explain 100% of the total system variability (Table S3—Supplementary Material). 

**Figure 7:**
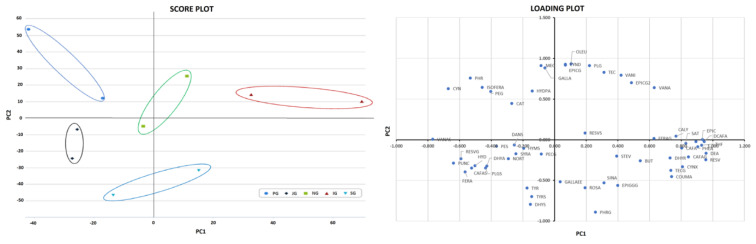
Score plots by multigroup analysis and loading plot for the first two components resulting from the principal component analysis performed using the pairwise jobs of the metabolomic data of the green tea cold brews.

The PCA score plot (Figure 7), where only the PC1 and PC2 eigenvalues are graphically represented, shows a clear separation of the different green tea brews prepared in hot water. In addition, the analysis of the loading plot (Figure 7) and of the squared cosines of the variables (Table S3—Supplementary Material) shows that resveratrol, theaflavin, delphinidin 3-O-arabinoside, caffeic acid 3-O-glucuronide, (-)-epicatechin, epicatechin 7-O-glucuronide, caffeic acid, cyanidin 3-O-xylosyl-rutinosidea, cyanidin and punicalin are the main components that discriminate the samples on the first coordinate (31.2%). The components that are important to the discrimination of tea samples on the second coordinate (25.1%) are instead oleuropein, cyanidin 3,5-O-diglucoside, (-)-epicatechin 3-O-gallate, pelargonidin, gallic acid, vanillin, phloretin, epicatechin 3′-O-glucuronide and tyrosol 4-sulfate.

Thereafter, in order to evaluate the ‘variable importance in projection’ of the PLS-DA model, the VIP approach was exploited to identify the compounds able to discriminate PGH vs. JGH, NGH, IGH, SGH and GGH tea brews. Considering these comparisons, 57 discriminant polyphenols were found as markers, mainly characterized by flavonoids and low-molecular-weight phenolics, that are reported in Table S4 (Supplementary Material) together with their VIP score (>1) and LogFC values (resulting from the fold change analysis; FC > 2). 

Overall, the LogFC values reported in Table S4 (Supplementary Material) can be considered indicative of the differences between the samples harvested in the different locations (i.e., Portugal, Jersey, Netherland, Italy, Switzerland and Germany). The phenolic markers showing the highest up-accumulation values for the different comparisons were PGH vs. JGH, theaflavin (LogFC = 5.16), caffeic acid (LogFC = 4.92) and caffeic acid 4-sulfate (LogFC = 5.24); PGH vs. NGH, (-)-epicatechin (LogFC = 3.98) and epigallocatechin 3-O-gallate-7-O-glucoside-4″-O-glucuronide (LogFC = 3.56); PGH vs. IGH, resveratrol (LogFC = 2.70), 2,4-dihydroxyacetophenone 5-sulfate (LogFC = 2.62), caffeic acid 3-O-glucuronide (LogFC = 2.46) and epigallocatechin 3-O-gallate-7-O-glucoside-4″-O-glucuronide (LogFC = 2.41); PGH vs. SGH, vanillic acid (LogFC = 2.84), resveratrol (LogFC = 2.63) and vanillin (LogFC = 2.56) and PGH vs. GGH, cyanidin (LogFC = 3.70) and p-coumaric acid (LogFC = 2.62).

## 4. Discussion

Numerous studies have been conducted pertaining to the chemical composition, quality and antioxidant capacities of teas grown in all corners of the globe (Africa, Asia, India, the Middle East, South America and Australia) [24,25,26,27,28,29,30]. However, such information regarding teas grown in Europe is very scarce. To the best of our knowledge, those that have been carried out were mainly on teas coming from the oldest tea plantation in Europe, in the Azores archipelago (Portugal), where the following information was obtained: phytochemicals content (such as theanine, theaflavin, catechins and amino acids), antioxidant properties in different plant parts and on processed green and white teas and the bioburden (bacteria and fungi) on bagged and loose green teas before and after boiling [31,32,33,34]. Regarding teas grown in mainland Europe, to the best of our knowledge, only two studies have been reported so far. One was on green tea from the Chà Camèlia plantation located on the northern Portuguese coast, where extracts of this tea were studied and compared to an Asian green tea extract for incorporation in whey protein films aimed at preserving Latin-style fresh cheeses [35]. The other study was related to the phytochemical profile and antioxidant properties of two green teas coming from two separate gardens located in northern Italy around the Lake Maggiore district [36], one of which (Compagnia del Lago Maggiore) was included in the present study. Hence, the need to address the knowledge gaps regarding the characterization of European teas is essential. 

Very recently, we conducted a study on the content of 15 elements (both potentially toxic and essential) in tea leaves collected from the same tea gardens reported here and found that there was no risk from the consumption of European teas for consumers in terms of potentially toxic elements [37]. This present investigation now builds on to this existing information regarding the characterization of European teas in terms of potential health benefits as assessed through their antioxidant profile, polyphenol content and metal chelating activity in both the more popular hot water brews and in the upcoming popularity of cold water brews. Cold tea brews are sometimes preferred due to their taste and unmatched flavor profile (less bitterness, caffeine and tannins) and are a popular beverage during summer months [38]. 

Amongst the European teas studied, no major differences concerning brewing conditions were observed, although in general hot brews tended to have higher antioxidant potential than cold brews due to the greater extraction efficiency of the polyphenolic compounds responsible for this activity, as already observed in other studies [39,40,41]. Concerning the types of tea from the different European countries, green teas showed greater TPC, TFC and antioxidant activity than the black and white ones, which is in accordance with the degree of oxidation of the teas [7,24,26]. It is well known that high catechin levels positively correlate with antioxidant activity, and these levels remain high in green teas because these polyphenols do not undergo oxidation. Instead, in black teas, the catechins are subjected to oxidation by their exposure to polyphenol oxidases and peroxidases that convert these compounds to dimeric and polymeric structures known as theaflavins and thearubigins, which are responsible for the characteristic aroma, flavor and color of black tea [7]. These compounds, however, account for the greater chelating ability observed in black teas compared to green teas, as reported here and in other studies [26,41]. Indeed, the specific moieties bearing adjacent hydroxyl and carbonyl functions, such as those found in theaflavins and thearubigins, have been suggested to play a role in the binding of transition metal ions involved in catalyzing free radical reactions [42]. This fact is well exemplified in the present study where the Portuguese green tea (PG), which had the highest TPC, TFC and antioxidant capacity amongst all the teas studied, showed the lowest metal chelating ability. White teas are generally considered unoxidized teas such as green teas; however, strictly speaking, this is incorrect. Since white teas do not undergo heat inactivation of enzymes, the so-called ‘killing the green’ halts the oxidation process. Therefore, catechins in white teas do undergo a certain degree of oxidation according to the processing methods used for withering and drying, and this is reflected in the levels of TPC, TFC and antioxidant capacity of the two white teas studied. Those related to the German white tea (GW) indeed fall between those of the black and green teas, whereas the levels observed for the white tea from the Azores (AW) are more similar to the black teas, indicating a greater extent of catechins oxidation. These observations are in accordance with our previous studies comparing green, black and white teas [24].

The comparison of our results on European teas with aqueous brews from other provenances is not exactly straightforward because of the high variability in assay conditions (incubation times), brewing techniques (temperature, time, water/leaf ratios typically ranging between 1–2.5%, shaking/stirring and leaf size), units of measurement for reporting data and cultivars used. Nevertheless, despite these drawbacks, the literature survey on tea brews shows that the levels of TPC, TFC, antioxidant capacity and metal chelating activity (MCA) of the European teas studied here fall within most of the ranges found by others and by us in similar studies performed on teas mostly from China, Africa and Taiwan. For this survey, when the literature data were not reported in mM equivalents as reported here, our data were converted for comparative purposes into either mg/mL by multiplying the concentration in mM by the molecular weight of the standard or in mg/g dry weight (DW) considering that 1 g of tea was brewed in 50 mL of water (mg/g DW = mg/L × 0.05 L/g).

In our first study on hot and cold brews obtained from black, white and green teas from China, TPC levels were in the range of 2.2–7.9 mM GAEq, which is within the TPC range found here (2.5–10.2 mM GAEq), ABTS values were in the range of 5–25 mM TXEq (present study 8–24.8 mM TXEq) and metal chelating activity values were in the range of 0.3–1.9 mM EDEq (present study, 0.34–0.4 mM EDEq) [43]. In a follow-up investigation on only hot brews of white, green and black teas from Africa (Malawi) [24] coming from the same cultivar, we found higher levels of TPC (18.1–23.6 mM GAEq vs. 3.8–10.2 mM GAEq, present study), ABTS values (34–55 mM TXEq vs. 10.3–24.8 mM TXEq, present study) and ORAC values (47–62 mM TXEq vs. 16.3–36 mM TXEq, present study); however, metal chelating activity values were very similar (0.18–0.48 mM EDEq vs. 0.34–0.40 mM EDEq, present study). In a further investigation on hot and cold brews of only white teas coming from China and Malawi, we found comparable values to those reported here (TPC: 1.43–7.63 mM GAEq vs. 4.4–5.5 mM GAEq, present study; TFC: 0.7–2.53 mM CEq vs. 0.70–0.78 mM CEq, present study; ABTS: 5.26–34.23 mM TXEq vs. 13.74–14.27 mM TXEq, present study; MCA: 0.3–0.6 mM EDEq vs. 0.34–0.36 mM EDEq, present study) [44]. Komes et al. studied several powdered, loose and bagged green teas purchased from specialized stores and prepared hot brews (80 °C/3 min). The results of the TPC found were in the range of 0.8–2.56 mg/mL GAEq, which is similar to our average value for hot green tea brews of 1.7 mg/mL GAEq [45]. Perez-Burillo et al. performed a study on hot brews (98 °C/7 min) of 19 green and 21 white teas, both loose and bagged, that were bought in Spanish supermarkets and found mean TPC values in white teas of 0.14 mg/mL GAEq vs. 0.9 mg/mL GAEq, which was found in our European teas; for green teas, they found mean values of 1.04 mg/mL GAEq vs. 1.7 mg/mL GAEq, which was found in the present study. Consequently, the antioxidant capacity that they found in the large sample of teas measured with the ABTS assay was also lower compared to the values found here (white teas: 7.23 mM TXEq vs. 14.27 mM TXEq, present study; green teas: 6.0 mM TXEq vs. 24.8 mM TXEq, present study) [46]. In 2019, Zhao et al. undertook a study on 30 hot brews (98 °C/5 min) from green, black and white Chinese teas with a high water/leaf ratio of 10%, and the mean TPC levels and ABTS values that were found fell within the range of those obtained from the European teas found here (TPC: 24–252 mg GAEq/g DW vs. 21–85 mg GAEq/g DW, present study; ABTS: 166–2532 mmol TXEq/g DW vs. 520–1240 mmol TXEq/g DW, present study) [30]. In a recent study conducted on hot brews (100 °C/10 min) of two green teas, each coming from two Italian gardens, Falla et al. found a TPC value for the green tea from Compagnia del Lago Maggiore of 8540 mg GAEq/100 g DW, which is close to the 7000 mg GAEq/100 g DW that we found for the same tea in this study, and an ABTS value of 623 mmol TXEq/g DW vs. 1020 mmol TXEq/g DW, present study [36]. Obviously, the differences observed are likely ascribable to the harvesting year, season and brewing techniques since the tea leaves came from the same tea garden and had the same processing method. Lin et al. prepared hot brews (100 °C/5 min) from green and black teas coming from a single estate in Taiwan, and the TPC values that they found for the green (0.92 mg/mL GAEq) and black teas (0.6 mg/mL GAEq) were similar to those found in the European teas (green = 1.7 mg/mL GAEq; black = 0.6 mg/mL GAEq). The ABTS values that they obtained were also close to ours (green = 1.02 mmol TXEq/g DW vs. 1.24 mmol TXEq/g DW, present study; black = 2.16 mmol TXEq/g DW vs. 0.52 mmol TXEq/g DW, present study) [26]. These same authors also performed a study on the properties of two types of green tea obtained from the same farm in Taiwan according to the brewing method. The TPC value they found for the cold brew (4 °C/24 h) was 0.74 mg/mL GAEq vs. the 1.44 mg/mL GAEq of the European teas studied here; for the hot brew (90 °C/20 min), the values were very similar to ours (1.86 mg/mL GAEq vs. 1.7 mg/mL GAEq, present study) [40]. Coe et al. studied the TPC of hot brews (90 °C/3 min) coming from 16 different commercially available brands of loose leaf teas bought in the UK, which included white, green and black teas, and they found that this was in the range of 20–95 mg/g GAEq DW [47]. The European teas fall well within this range (30–85 mg/g GAEq DW). The mean ABTS, ORAC and TPC values (10.3 mM TXEq; 16.3 mM TXEq; 0.6 mg/mL GAEq, respectively) of the European black teas studied here also fall within or close to the range of values found in a study on hot brews (100 °C/1 min) of 7 different bagged black tea brands present on the British market (ABTS = 9.6–13.4 mM TXEq; ORAC = 7.4–13 mM TXEq; 0.95–1.25 mg/mL GAEq) [48]. 

To further characterize the European teas, UV-Vis spectroscopy was used as a simple, rapid, stable and accurate quantitative tool for the simultaneous identification of teas according to their variety (black, white or green tea). Indeed, the PCA statistical treatment based on UV-Vis data was successfully able to discriminate green, black and white European tea brews prepared according to the standard preparation of a homemade cup of tea, both for hot brews and for cold ones, but not their origin as reported by others [49]. This is the first report on UV-Vis spectroscopy of cold tea brews, as previous studies were performed only on hot brews of green and black teas from Argentina, Brazil, Sri Lanka, China and Africa [50,51,52]. The results obtained are consistent with the aforementioned studies demonstrating the clustering of tea varieties based on the UV-Vis data, and thus confirming the processing methods adopted by European tea growers for obtaining their tea varieties.

Metabolomics is increasingly being used as a valuable technique for determining unbiased, phytochemical variations between tea samples of different origins, tea products or teas that have been processed differently, as recently reviewed by Farag et al. [53]. Indeed, the PCA multivariate statistical approach of the metabolomics data set obtained for the first time on European tea infusions was able to show explicit differences amongst the hot green tea brews, discriminating them according to their origin. Several chemical features, including resveratrol, theaflavin, (-)-epicatechin, oleuropein, (-)-epicatechin 3-O-gallate and punicalin, were identified as potential markers that differentiated these teas. Furthermore, the VIP selection method was able to show that there were 57 discriminant polyphenols responsible for the variations between the green tea from Portugal and those from the other countries.

## 5. Conclusions

Overall, our findings demonstrate for the first time that teas grown in Europe are good quality teas endowed with levels of health-promoting polyphenols and flavonoids and antioxidant capacities similar to those grown in other parts of the world, suggesting that European teas represent a valid alternative to the more common Chinese, Indian and African teas. This research is an important contribution to the characterization of European teas, providing essential and important information that could be of interest to both European tea growers and consumers in terms of guidance and support for the selection of teas grown in the old continent, along with the best brewing conditions for maximizing the health benefits of tea. Furthermore, our findings may have significant implications regarding the quality of tea planted in Europe and on the feasibility of establishing tea plantations in this region. An additional study is now underway with European teas where the plucking period and processing techniques are being harmonized between growers in order to have more comparable results amongst the different tea gardens. Soil samples from the tea gardens will also be analyzed for metal analysis uptake to be correlated with those found in the leaves and infusions.

## Figures and Tables

**Table 1:** List of teas. Tea garden name, home country, tea type and label of the European teas studied.

European Garden	Country	Type	Label
Het Zuyderblad	The Netherlands	Black	NB
		Green	NG
Compagnia del Lago Maggiore	Italy	Black	IB
		Green	IG
Tschanara Teagarden	Germany	Black	GB
		Green	GG
		White	GW
Casa del Tè Monte Verità	Switzerland	Black	SB
		Green	SG
Jersey Fine Tea	Jersey (UK)	Black	JB
		Green	JG
Chà Camèlia	Portugal	Green	PG
Agrarian Devt. Services Sao Miguel	Azores (Portugal)	White	AW

**Table 2:** Total phenolic content (TPC), total flavonoid content (TFC), antioxidant activity data (ORAC, ABTS, FRAP) and metal chelating activity (MCA) of the tea brews studied. Samples are grouped by type of brew and type of tea, and the means of each group is reported in bold. Letters within each column indicate homogeneous sub-classes resulting from Tukey’s post hoc multiple comparison tests (*p* < 0.05) performed between all samples (uppercase) and between means of data (lowercase). Statistical differences between samples of each tea type (black, green, white) are reported in Table S5.

Code	mM GAEq	mM CEq	mM TXEq	mM TXEq	mM AAEq	mM EDEq
***Cold***						
NBC	1.3 ± 0.1 M	0.21 ± 0.02 N	10.5 ± 1.4 JK	5.3 ± 0.7 O	2.4 ± 0.4 K	0.39 ± 0.03 CDEF
IBC	2.0 ± 0.2 LM	0.32 ± 0.02 M	11.3 ± 1.2 IJK	7.1 ± 0.9 NO	3.9 ± 0.6 J	0.35 ± 0.03 FGHI
GBC	2.4 ± 0.1 KL	0.36 ± 0.02 LM	13.3 ± 0.6 IJK	7.7 ± 1.3 MN	3.5 ± 0.6 JK	0.40 ± 0.04 BCDE
SBC	2.8 ± 0.3 JK	0.40 ± 0.02 L	13.7 ± 1.4 IJ	9.4 ± 1.4 KLM	5.3 ± 0.7 HI	0.41 ± 0.03 ABC
JBC	3.8 ± 0.4 HI	0.59 ± 0.06 JK	19.4 ± 2.3 H	10.5 ± 1.1 KL	6.3 ± 0.7 GH	0.44 ± 0.02 A
black	2.5 ± 0.9 z	0.4 ± 0.1 z	13.6 ± 3.5 y	8.0 ± 2.0 z	4.3 ± 1.5 z	0.40 ± 0.03 x
NGC	6.9 ± 0.4 F	1.01 ± 0.09 H	30.8 ± 3.4 EF	18.4 ± 2.4 HI	13.7 ± 1.7 E	0.33 ± 0.03 I
IGC	7.1 ± 0.5 F	1.14 ± 0.07 FG	30.0 ± 2.2 F	19.0 ± 1.8 GHI	14.0 ± 1.5 DE	0.43 ± 0.03 AB
GGC	8.9 ± 0.3 DE	1.42 ± 0.12 C	33.2 ± 2.5 CDEF	21.8 ± 3.1 EF	17.5 ± 1.8 B	0.34 ± 0.02 HI
SGC	8.9 ± 0.9 DE	1.22 ± 0.11 EF	31.6 ± 4.0 DEF	22.4 ± 1.7 DEF	18.0 ± 2.9 B	0.45 ± 0.03 A
JGC	8.7 ± 0.5 DE	1.29 ± 0.09 DE	33.5 ± 1.7 CDEF	22.1 ± 2.4 EF	16.1 ± 1.9 C	0.39 ± 0.03 CDEF
PGC	10.5 ± 0.8 B	1.61 ± 0.09 B	40.5 ± 3.6 AB	26.2 ± 1.8 BC	21.8 ± 2.4 A	0.10 ± 0.02 L
green	8.5 ± 1.3 xy	1.3 ± 0.2 xy	33.3 ± 3.8 x	21.7 ± 2.8 xy	16.9 ± 3.0 x	0.34 ± 0.13 x
AWC	1.6 ± 0.1 LM	0.30 ± 0.02 M	9.6 ± 0.5 K	6.9 ± 0.7 NO	4.0 ± 0.5 IJ	0.29 ± 0.02 J
GWC	7.1 ± 0.4 F	1.10 ± 0.05 G	26.5 ± 1.8 G	20.6 ± 2.2 FG	15.4 ± 2.0 CD	0.38 ± 0.02 CDEFG
white	4.4 ± 3.9 yz	0.70 ± 0.56 yz	18.03 ± 11.9 y	13.74 ± 9.7 yz	9.7 ± 8.1 xyz	0.34 ± 0.07 x
*Hot*						
NBH	2.4 ± 0.6 KL	0.32 ± 0.06 M	11.8 ± 3.1 IJK	7.2 ± 2.4 NO	3.1 ± 0.6 JK	0.40 ± 0.03 BCDE
IBH	3.5 ± 0.6 IJ	0.43 ± 0.07 L	14.3 ± 2.2 I	10.2 ± 1.1 KL	4.3 ± 0.4 IJ	0.38 ± 0.04 CDEFG
GBH	3.7 ± 0.2 HI	0.52 ± 0.04 K	14.7 ± 1.1 I	9.2 ± 0.7 LM	4.4 ± 0.3 IJ	0.39 ± 0.04 BCDEF
SBH	4.8 ± 0.9 G	0.63 ± 0.08 J	19.5 ± 2.1 H	13.6 ± 1.8 J	6.7 ± 0.9 G	0.41 ± 0.02 ABCD
JBH	4.5 ± 0.3 G	0.63 ± 0.04 J	21.2 ± 4.2 H	11.3 ± 0.7 K	6.1 ± 0.4 GH	0.40 ± 0.02 BCDE
black	3.8 ± 1.0 z	0.5 ± 0.13 z	16.3 ± 3.9 y	10.3 ± 2.4 z	4.9 ± 1.5 z	0.40 ± 0.01 x
NGH	9.7 ± 0.9 C	1.27 ± 0.09 DE	36.2 ± 2.9 BC	24.4 ± 1.8 CD	15.6 ± 1.3 C	0.37 ± 0.03 EFGH
IGH	8.3 ± 0.8 E	1.12 ± 0.10 G	35.2 ± 4.5 CD	20.4 ± 1.2 FGH	13.4 ± 1.8 E	0.37 ± 0.03 EFGH
GGH	9.0 ± 0.7 D	1.30 ± 0.09 D	30.4 ± 1.7 EF	22.2 ± 2.7 EF	15.2 ± 1.1 CD	0.35 ± 0.03 GHI
SGH	10.9 ± 0.7 B	1.43 ± 0.09 C	36.2 ± 3.8 C	26.9 ± 1.8 B	18.8 ± 1.3 B	0.41 ± 0.02 ABC
JGH	9.4 ± 0.7 CD	1.28 ± 0.10 DE	34.1 ± 4.6 CDE	23.7 ± 2.7 DE	14.2 ± 1.5 DE	0.35 ± 0.03 FGHI
PGH	13.8 ± 1.1 A	1.84 ± 0.11 A	44.1 ± 5.7 A	31.1 ± 3.7 A	21.7 ± 2.1 A	0.17 ± 0.03 K
green	10.2 ± 2.0 x	1.4 ± 0.25 xy	36.0 ± 4.5 x	24.8 ± 3.8 x	16.5 ± 3.1 xy	0.34 ± 0.08 x
AWH	4.2 ± 0.2 GH	0.64 ± 0.05 J	15.2 ± 1.1 I	10.9 ± 0.7 KL	6.0 ± 0.6 GH	0.37 ± 0.03 DEFG
GWH	6.7 ± 0.5 F	0.92 ± 0.06 I	21.7 ± 0.9 H	17.6 ± 1.2 I	11.5 ± 1.0 F	0.34 ± 0.03 HI
white	5.5 ± 1.7 yz	0.78 ± 0.19 yz	18.44 ± 4.6 y	14.27 ± 4.7 yz	8.7 ± 3.9 xyz	0.36 ± 0.02 x

**Table 3:** Matrix of Pearson’s correlation coefficient (a) and (b) relative *p*-values.

**(a)**		**TPC**	**TFC**	**ORAC**	**ABTS**	**FRAP**	**MCA**
	TPC	1	0.988	0.976	0.993	0.964	−0.419
	TFC	0.988	1	0.977	0.987	0.982	−0.449
	ORAC	0.976	0.977	1	0.975	0.961	−0.409
	ABTS	0.993	0.987	0.975	1	0.977	−0.403
	FRAP	0.964	0.982	0.961	0.977	1	−0.439
	MCA	−0.419	−0.449	−0.409	−0.403	−0.439	1
**(b)**		**TPC**	**TFC**	**ORAC**	**ABTS**	**FRAP**	**MCA**
	TPC	0	<0.0001	<0.0001	<0.0001	<0.0001	0.033
	TFC	<0.0001	0	<0.0001	<0.0001	<0.0001	0.021
	ORAC	<0.0001	<0.0001	0	<0.0001	<0.0001	0.038
	ABTS	<0.0001	<0.0001	<0.0001	0	<0.0001	0.041
	FRAP	<0.0001	<0.0001	<0.0001	<0.0001	0	0.025
	MCA	0.033	0.021	0.038	0.041	0.025	0

Values are all different from 0 with a significance alpha level of 0.05.

## Data Availability

Data supporting the reported results can be provided upon request to the corresponding author.

## Supplementary Materials

The following supporting information can be downloaded at https://www.mdpi.com/article/10.3390/antiox12061306/s1; Table S1: Eigenvalues, variance explained and the contribution of the variables (% Squared cosines) for the first two principal components extracted resulting from the principal component analysis performed using the UV-Vis data (320–500 nm, Δλ = 5 nm) of the (a) cold brews and (b) hot brews; Table S2: Phenolic compounds putatively annotated in the green tea hot brews using a high-resolution untargeted UHPLC-MS approach with their abbreviations; Table S3: Eigenvalues, variance explained and the contribution of the variables (% Squared cosines) for the first two principal components extracted resulting from the principal component analysis performed using the phenolic compounds putatively annotated in the green tea hot brews; Table S4: Compounds able to discriminate PGH vs. JGH, NGH, IGH, SGH and GGH tea hot brews with a PLS-DA model. VIP score (>1) and LogFC values (resulting from the fold change analysis; FC > 2); Table S5: Total phenolic content (TPC), total flavonoid content (TFC), antioxidant activity data (ORAC, ABTS, FRAP) and metal chelating activity (MCA) of the studied tea brews with statistical differences resulting from Tukey’s post hoc multiple comparison tests (*p* < 0.05) performed with tea samples of the same type (black, green, white).
