# Supplementary material for: Comparative Analysis of Hot and Cold Brews from Single-Estate Teas (Camellia sinensis) Grown across Europe: An Emerging Specialty Product
Source: Antioxidants (Basel). 2023 Jun 20;12(6):1306. doi: 10.3390/antiox12061306 (PMC10295353; doi:10.3390/antiox12061306)
Supplement: Supplementary file 1 [file antioxidants-12-01306-s001.zip › antioxidants-2450080-supplementary-6.20.docx]

**Table S1.** Eigenvalues, variance explained and the contribution of the variables (% Squared cosines) for the first two principal components extracted resulting from the principal component analysis performed using the UV-Vis data (320-500 nm, Δλ = 5 nm) of the **a)** cold brews and **b)** hot brews.

|  | **a) Cold Brews** | | **b) Hot Brews** | |
| --- | --- | --- | --- | --- |
|  | **F1** | **F2** | **F1** | **F2** |
| Eigenvalue | 24.074 | 11.860 | 28.041 | 8.443 |
| Variability (%) | 65.064 | 32.053 | 75.787 | 22.820 |
| Cumulative % | 65.064 | 97.117 | 75.787 | 98.606 |
| **Variables** | **Squared cosines of the variables** | | | |
| 320 | 0.393 | **0.558** | 0.429 | **0.506** |
| 325 | 0.428 | **0.541** | 0.478 | **0.495** |
| 330 | 0.437 | **0.544** | 0.485 | **0.500** |
| 335 | 0.430 | **0.559** | 0.483 | **0.508** |
| 340 | 0.426 | **0.568** | 0.482 | **0.513** |
| 345 | 0.425 | **0.569** | 0.485 | **0.510** |
| 350 | 0.428 | **0.565** | 0.494 | **0.500** |
| 355 | 0.433 | **0.558** | **0.510** | 0.482 |
| 360 | 0.440 | **0.551** | **0.535** | 0.455 |
| 365 | 0.452 | **0.539** | **0.576** | 0.415 |
| 370 | 0.475 | **0.518** | **0.638** | 0.354 |
| 375 | **0.510** | 0.483 | **0.719** | 0.274 |
| 380 | **0.566** | 0.427 | **0.810** | 0.181 |
| 385 | **0.653** | 0.338 | **0.899** | 0.089 |
| 390 | **0.765** | 0.220 | **0.959** | 0.026 |
| 395 | **0.881** | 0.095 | **0.981** | 0.001 |
| 400 | **0.951** | 0.012 | **0.974** | 0.007 |
| 405 | **0.949** | 0.007 | **0.951** | 0.031 |
| 410 | **0.896** | 0.055 | **0.926** | 0.058 |
| 415 | **0.838** | 0.116 | **0.905** | 0.081 |
| 420 | **0.791** | 0.167 | **0.888** | 0.100 |
| 425 | **0.755** | 0.207 | **0.874** | 0.117 |
| 430 | **0.725** | 0.241 | **0.860** | 0.132 |
| 435 | **0.709** | 0.261 | **0.848** | 0.145 |
| 440 | **0.703** | 0.275 | **0.842** | 0.153 |
| 445 | **0.708** | 0.278 | **0.841** | 0.156 |
| 450 | **0.719** | 0.274 | **0.842** | 0.157 |
| 455 | **0.734** | 0.264 | **0.844** | 0.155 |
| 460 | **0.741** | 0.252 | **0.845** | 0.152 |
| 465 | **0.740** | 0.246 | **0.843** | 0.151 |
| 470 | **0.732** | 0.246 | **0.837** | 0.154 |
| 475 | **0.727** | 0.243 | **0.835** | 0.154 |
| 480 | **0.723** | 0.230 | **0.832** | 0.150 |
| 485 | **0.720** | 0.214 | **0.831** | 0.144 |
| 490 | **0.707** | 0.208 | **0.830** | 0.139 |
| 495 | **0.693** | 0.208 | **0.820** | 0.144 |
| 500 | **0.670** | 0.223 | **0.810** | 0.154 |
| *Values in bold correspond for each variable to the factor for which the squared cosine is the largest* | | | | |

**Table S2**. Phenolic compounds putatively annotated in the green tea hot brews using a high-resolution untargeted UHPLC-MS approach with their abbreviation.

| **Class and Name** | **Abbreviation** |
| --- | --- |
| **FLAVONOIDS** |  |
| *ANTHOCYANINS* |  |
| 4-O-Methyldelphinidin 3-O-D-glucoside | MEG |
| Cyanidin | CYN |
| Cyanidin 3,5-O-diglucoside | CYND |
| Cyanidin 3-O-xylosyl-rutinosidea | CYNX |
| Delphinidin 3-O-arabinoside | DEA |
| Pelargonidin | PLG |
| Pelargonidin 3-O-sophoroside | PLGS |
| Peonidin 3-O-(2-O-(6-O-(E)-caffeoyl-D-glucosyl)-D-glucoside)-5-O-D-glucoside | PECG |
| Peonidin 3-O-sophoroside | PES |
| Petunidin 3-O-glucoside | PEG |
| *DIHYDROCHALCONES* |  |
| Phloretin | PHR |
| Phloretin 2'-O-glucuronide | PHRG |
| *FLAVANOLS* |  |
| (+)-Catechin | CAT |
| (-)-Epicatechin | EPIC |
| (-)-Epicatechin 3-O-gallate | EPICG |
| Epicatechin 3'-O-glucuronide | EPICG2 |
| Epicatechin 7-O-glucuronide | EPICG3 |
| Epigallocatechin 3-O-gallate-7-O-glucoside-4''-O-glucuronide | EPIGGG |
| Theaflavin | THF |
| *ISOFLAVONOIDS* |  |
| Butin | BUT |
| Calycosin | CALY |
| Sativanone | SAT |
| Stevenin | STEV |
| Tectoridin | TEC |
| Tectorigenin | TECG |
| **PHENOLIC ACIDS** |  |
| *HYDROXYBENZOIC ACIDS* |  |
| Gallic acid | GALLA |
| Gallic acid ethyl ester | GALLAEE |
| Punicalin | PUNC |
| Syringic acid | SYRA |
| Vanillic acid | VANA |
| Vanillic acid 4-sulfate | VANAS |
| *HYDROXYCINNAMIC ACIDS* |  |
| 4,5-Dicaffeoylquinic acid | DCAFA |
| Caffeic acid | CAFA |
| Caffeic acid 3-O-glucuronide | CAFAG |
| Caffeic acid 4-sulfate | CAFAS |
| Ferulic acid | FERA |
| Ferulic acid 4-O-glucuronide | FERAG |
| Isoferulic acid | ISOFERA |
| p-Coumaric acid | COUMA |
| Rosmarinic acid | ROSA |
| Sinapic acid | SINA |
| *HYDROXYPHENYLPROPANOIC ACIDS* |  |
| 3-Phenylpropionic acid | PHEA |
| 4-Hydroxyphenyl-2-propionic acid | HYDPA |
| Danshensu | DANS |
| Dihydrosinapic acid | DHYA |
| Hydroxydanshensu | HYD |
| **STILBENES** |  |
| Dihydroresveratrol | DHYR |
| Resveratrol | RESV |
| Resveratrol 3-sulfate | RESVS |
| trans-Resveratrol 4'-O-glucuronide | RESVG |
| **OTHER POLYPHENOLS** |  |
| Vanillin | VANI |
| 2,4-Dihydroxyacetophenone 5-sulfate | DHYS |
| 2-Hydroxy-4-methoxyacetophenone 5-sulfate | HYMS |
| Norathyriol | NORT |
| Oleuropein | OLEU |
| Tyrosol | TYR |
| Tyrosol 4-sulfate | TYRS |

**Table S3.** Eigenvalues, variance explained and the contribution of the variables (% Squared cosines) for the first two principal components extracted resulting from the principal component analysis performed using the phenolic compounds putatively annotated in the green tea hot brews.

|  | **PC1** | **PC2** |
| --- | --- | --- |
| Eigenvalue | 17.807 | 14.303 |
| Variability (%) | 31.241 | 25.093 |
| Cumulative % | 31.241 | 56.334 |
| **Squared cosines of the variables:** | | |
| MEG | 0.0074 | 0.8331 |
| CYN | 0.4514 | 0.3962 |
| CYND | 0.0046 | 0.8591 |
| CYNX | 0.6529 | 0.1099 |
| DEA | 0.8637 | 0.0047 |
| PLG | 0.0485 | 0.8297 |
| PLGS | 0.1825 | 0.1042 |
| PECG | 0.0071 | 0.0309 |
| PES | 0.1353 | 0.0066 |
| PEG | 0.1629 | 0.3519 |
| PHR | 0.2845 | 0.5761 |
| PHRG | 0.0663 | 0.7910 |
| CAT | 0.0729 | 0.1989 |
| EPIC | 0.7973 | 0.0006 |
| EPICG | 0.0046 | 0.8448 |
| EPICG2 | 0.2358 | 0.4901 |
| EPICG3 | 0.7162 | 0.0436 |
| EPIGGG | 0.1589 | 0.3135 |
| THF | 0.8942 | 0.0004 |
| BUT | 0.2933 | 0.0669 |
| CALY | 0.5876 | 0.0021 |
| SAT | 0.6448 | 0.0105 |
| STEV | 0.1544 | 0.0408 |
| TEC | 0.0969 | 0.6887 |
| TECG | 0.5395 | 0.1423 |
| GALLA | 0.0038 | 0.7816 |
| GALLAEE | 0.0013 | 0.2678 |
| PUNC | 0.4074 | 0.0829 |
| SYRA | 0.0596 | 0.0299 |
| VANA | 0.3970 | 0.4100 |
| VANAS | 0.5932 | 0.0000 |
| DCAFA | 0.8722 | 0.0000 |
| CAFA | 0.6879 | 0.0021 |
| CAFAG | 0.8183 | 0.0073 |
| CAFAS | 0.2745 | 0.1214 |
| FERA | 0.3195 | 0.1563 |
| FERAG | 0.3938 | 0.0003 |
| ISOFERA | 0.2089 | 0.4157 |
| COUMA | 0.5465 | 0.2046 |
| ROSA | 0.0368 | 0.3467 |
| SINA | 0.0975 | 0.2797 |
| PHEA | 0.9148 | 0.0278 |
| HYDPA | 0.0201 | 0.3600 |
| DANS | 0.0646 | 0.0040 |
| DHYA | 0.1898 | 0.1215 |
| HYD | 0.2532 | 0.1010 |
| DHYR | 0.5326 | 0.0500 |
| RESV | 0.9118 | 0.0608 |
| RESVS | 0.0371 | 0.0070 |
| RESVG | 0.3500 | 0.0545 |
| VANI | 0.1763 | 0.6319 |
| DHYS | 0.0231 | 0.6306 |
| HYMS | 0.0384 | 0.0110 |
| NORT | 0.0851 | 0.0539 |
| OLEU | 0.0108 | 0.8729 |
| TYR | 0.0305 | 0.3516 |
| TYRS | 0.0209 | 0.4879 |

**Table S4.** Compounds able to discriminate PGH vs JGH, NGH, IGH, SGH and GGH tea hot brews with a PLS-DA model. VIP score (> 1) and LogFC values (resulting from Fold-Change analysis; FC > 2).

**Table S5.** Total phenolic content (TPC), Total flavonoid content (TFC), antioxidant activity data (ORAC, ABTS, FRAP) and metal chelating activity (MCA) of the studied tea brews with statistical differences resulting from Tukey’s post hoc multiple comparison test (p<0.05) performed between tea samples of the same type (black, green, white).

| **Code** | **TPC (mM GAEq)** | | | |  | **TFC (mM CEq)** | | | |  | **ORAC (mM TXEq)** | | | |  | **ABTS (mM TXEq)** | | | |  | **FRAP (mM AAEq)** | | | |  | **MCA (mM EDEq)** | | | |
| --- | --- | --- | --- | --- | --- | --- | --- | --- | --- | --- | --- | --- | --- | --- | --- | --- | --- | --- | --- | --- | --- | --- | --- | --- | --- | --- | --- | --- | --- |
| ***Cold*** |  |  |  |  |  |  |  |  |  |  |  |  |  |  |  |  |  |  |  |  |  |  |  |  |  |  |  |  |  |
| NBC | 1.3 | ± | 0.1 | E |  | 0.21 | ± | 0.02 | F |  | 10.5 | ± | 1.4 | E |  | 5.3 | ± | 0.7 | F |  | 2.4 | ± | 0.4 | G |  | 0.39 | ± | 0.03 | B |
| IBC | 2.0 | ± | 0.2 | D |  | 0.32 | ± | 0.02 | E |  | 11.3 | ± | 1.2 | DE |  | 7.1 | ± | 0.9 | E |  | 3.9 | ± | 0.6 | DE |  | 0.35 | ± | 0.03 | C |
| GBC | 2.4 | ± | 0.1 | CD |  | 0.36 | ± | 0.02 | DE |  | 13.3 | ± | 0.6 | BCD |  | 7.7 | ± | 1.3 | E |  | 3.5 | ± | 0.6 | EF |  | 0.40 | ± | 0.04 | B |
| SBC | 2.8 | ± | 0.3 | C |  | 0.40 | ± | 0.02 | CD |  | 13.7 | ± | 1.4 | BCD |  | 9.4 | ± | 1.4 | CD |  | 5.3 | ± | 0.7 | C |  | 0.41 | ± | 0.03 | AB |
| JBC | 3.8 | ± | 0.4 | B |  | 0.59 | ± | 0.06 | A |  | 19.4 | ± | 2.3 | A |  | 10.5 | ± | 1.1 | BC |  | 6.3 | ± | 0.7 | AB |  | 0.44 | ± | 0.02 | A |
| **black** | **2.5** | ± | **0.9** | **z** |  | **0.4** | ± | **0.1** | **z** |  | **13.6** | ± | **3.5** | **y** |  | **8.0** | ± | **2.0** | **z** |  | **4.3** | ± | **1.5** | **z** |  | **0.40** | ± | **0.03** | **x** |
| NGC | 6.9 | ± | 0.4 | G |  | 1.01 | ± | 0.09 | F |  | 30.8 | ± | 3.4 | E |  | 18.4 | ± | 2.4 | G |  | 13.7 | ± | 1.7 | FG |  | 0.33 | ± | 0.03 | G |
| IGC | 7.1 | ± | 0.5 | G |  | 1.14 | ± | 0.07 | E |  | 30.0 | ± | 2.2 | E |  | 19.0 | ± | 1.8 | G |  | 14.0 | ± | 1.5 | EFG |  | 0.43 | ± | 0.03 | AB |
| GGC | 8.9 | ± | 0.3 | EF |  | 1.42 | ± | 0.12 | C |  | 33.2 | ± | 2.5 | CDE |  | 21.8 | ± | 3.1 | EF |  | 17.5 | ± | 1.8 | BC |  | 0.34 | ± | 0.02 | FG |
| SGC | 8.9 | ± | 0.9 | EF |  | 1.22 | ± | 0.11 | DE |  | 31.6 | ± | 4.0 | DE |  | 22.4 | ± | 1.7 | DEF |  | 18.0 | ± | 2.9 | B |  | 0.45 | ± | 0.03 | A |
| JGC | 8.7 | ± | 0.5 | EF |  | 1.29 | ± | 0.09 | D |  | 33.5 | ± | 1.7 | CDE |  | 22.1 | ± | 2.4 | EF |  | 16.1 | ± | 1.9 | CD |  | 0.39 | ± | 0.03 | CD |
| PGC | 10.5 | ± | 0.8 | BC |  | 1.61 | ± | 0.09 | B |  | 40.5 | ± | 3.6 | AB |  | 26.2 | ± | 1.8 | BC |  | 21.8 | ± | 2.4 | A |  | 0.10 | ± | 0.02 | I |
| **green** | **8.5** | ± | **1.3** | **xy** |  | **1.3** | ± | **0.2** | **xy** |  | **33.3** | ± | **3.8** | **x** |  | **21.7** | ± | **2.8** | **xy** |  | **16.9** | ± | **3.0** | **x** |  | **0.34** | ± | **0.13** | **x** |
| AWC | 1.6 | ± | 0.1 | D |  | 0.30 | ± | 0.02 | D |  | 9.6 | ± | 0.5 | D |  | 6.9 | ± | 0.7 | D |  | 4.0 | ± | 0.5 | D |  | 0.29 | ± | 0.02 | C |
| GWC | 7.1 | ± | 0.4 | A |  | 1.10 | ± | 0.05 | A |  | 26.5 | ± | 1.8 | A |  | 20.6 | ± | 2.2 | A |  | 15.4 | ± | 2.0 | A |  | 0.38 | ± | 0.02 | A |
| **white** | **4.4** | ± | **3.9** | **yz** |  | **0.70** | ± | **0.56** | **yz** |  | **18.03** | ± | **11.9** | **y** |  | **13.74** | ± | **9.7** | **yz** |  | **9.7** | ± | **8.1** | **xyz** |  | **0.34** | ± | **0.07** | **x** |
| ***Hot*** |  |  |  |  |  |  |  |  |  |  |  |  |  |  |  |  |  |  |  |  |  |  |  |  |  |  |  |  |  |
| NBH | 2.4 | ± | 0.6 | CD |  | 0.32 | ± | 0.06 | E |  | 11.8 | ± | 3.1 | CDE |  | 7.2 | ± | 2.4 | E |  | 3.1 | ± | 0.6 | F |  | 0.40 | ± | 0.03 | B |
| IBH | 3.5 | ± | 0.6 | B |  | 0.43 | ± | 0.07 | C |  | 14.3 | ± | 2.2 | BC |  | 10.2 | ± | 1.1 | BCD |  | 4.3 | ± | 0.4 | D |  | 0.38 | ± | 0.04 | BC |
| GBH | 3.7 | ± | 0.2 | B |  | 0.52 | ± | 0.04 | B |  | 14.7 | ± | 1.1 | B |  | 9.2 | ± | 0.7 | D |  | 4.4 | ± | 0.3 | D |  | 0.39 | ± | 0.04 | B |
| SBH | 4.8 | ± | 0.9 | A |  | 0.63 | ± | 0.08 | A |  | 19.5 | ± | 2.1 | A |  | 13.6 | ± | 1.8 | A |  | 6.7 | ± | 0.9 | A |  | 0.41 | ± | 0.02 | AB |
| JBH | 4.5 | ± | 0.3 | A |  | 0.63 | ± | 0.04 | A |  | 21.2 | ± | 4.2 | A |  | 11.3 | ± | 0.7 | B |  | 6.1 | ± | 0.4 | B |  | 0.40 | ± | 0.02 | B |
| **black** | **3.8** | ± | **1.0** | **z** |  | **0.5** | ± | **0.13** | **z** |  | **16.3** | ± | **3.9** | **y** |  | **10.3** | ± | **2.4** | **z** |  | **4.9** | ± | **1.5** | **z** |  | **0.40** | ± | **0.01** | **x** |
| NGH | 9.7 | ± | 0.9 | CD |  | 1.27 | ± | 0.09 | D |  | 36.2 | ± | 2.9 | BC |  | 24.4 | ± | 1.8 | CD |  | 15.6 | ± | 1.3 | DE |  | 0.37 | ± | 0.03 | DEF |
| IGH | 8.3 | ± | 0.8 | F |  | 1.12 | ± | 0.10 | E |  | 35.2 | ± | 4.5 | CD |  | 20.4 | ± | 1.2 | FG |  | 13.4 | ± | 1.8 | G |  | 0.37 | ± | 0.03 | DE |
| GGH | 9.0 | ± | 0.7 | E |  | 1.30 | ± | 0.09 | D |  | 30.4 | ± | 1.7 | E |  | 22.2 | ± | 2.7 | EF |  | 15.2 | ± | 1.1 | DEF |  | 0.35 | ± | 0.03 | EFG |
| SGH | 10.9 | ± | 0.7 | B |  | 1.43 | ± | 0.09 | C |  | 36.2 | ± | 3.8 | BC |  | 26.9 | ± | 1.8 | B |  | 18.8 | ± | 1.3 | B |  | 0.41 | ± | 0.02 | BC |
| JGH | 9.4 | ± | 0.7 | DE |  | 1.28 | ± | 0.10 | D |  | 34.1 | ± | 4.6 | CDE |  | 23.7 | ± | 2.7 | CDE |  | 14.2 | ± | 1.5 | EFG |  | 0.35 | ± | 0.03 | EFG |
| PGH | 13.8 | ± | 1.1 | A |  | 1.84 | ± | 0.11 | A |  | 44.1 | ± | 5.7 | A |  | 31.1 | ± | 3.7 | A |  | 21.7 | ± | 2.1 | A |  | 0.17 | ± | 0.03 | H |
| **green** | **10.2** | ± | **2.0** | **x** |  | **1.4** | ± | **0.25** | **xy** |  | **36.0** | ± | **4.5** | **x** |  | **24.8** | ± | **3.8** | **x** |  | **16.5** | ± | **3.1** | **xy** |  | **0.34** | ± | **0.08** | **x** |
| AWH | 4.2 | ± | 0.2 | C |  | 0.64 | ± | 0.05 | C |  | 15.2 | ± | 1.1 | C |  | 10.9 | ± | 0.7 | C |  | 6.0 | ± | 0.6 | C |  | 0.37 | ± | 0.03 | A |
| GWH | 6.7 | ± | 0.5 | B |  | 0.92 | ± | 0.06 | B |  | 21.7 | ± | 0.9 | B |  | 17.6 | ± | 1.2 | B |  | 11.5 | ± | 1.0 | B |  | 0.34 | ± | 0.03 | B |
| **white** | **5.5** | ± | **1.7** | **yz** |  | **0.78** | ± | **0.19** | **yz** |  | **18.44** | ± | **4.6** | **y** |  | **14.27** | ± | **4.7** | **yz** |  | **8.7** | ± | **3.9** | **xyz** |  | **0.36** | ± | **0.02** | **x** |
